# Bioinspired Intelligence II

**DOI:** 10.3390/biomimetics7020076

**Published:** 2022-06-09

**Authors:** Juan Luis Crespo-Mariño, Andrés Segura-Castillo

**Affiliations:** 1 LIANA Lab (IA Lab for Natural Sciences), Department of Mechatronics Engineering, Instituto Tecnológico de Costa Rica, Cartago 30101, Costa Rica; 2 Technological Research and Innovation Laboratory (LIIT), Vicerrectoría de Investigación, Universidad Estatal a Distancia (UNED), San José 474-2050, Costa Rica

Bioinspired intelligence methods are currently common approaches for both engineers and the scientists. The growing and economically feasible availability of high-performance computing platforms, massive storage capacities, and high-speed networks have contributed significantly to this. Furthermore, along with these technological advances, a vibrant practitioner community is growing and contributing innovative software developments towards the modeling of complex issues, which are otherwise impossible to treat by means of classical or analytical paradigms. Thus, the implementation of biologically inspired models makes sense and has been widely adopted as a viable alternative to reduce complexity.

It is with this perspective in mind that this Special Issue was created. The works published show multi- and transdisciplinary efforts that present innovative findings beyond specific knowledge areas. We expect that these contributions will give readers insights on new methods and technologies for their own bioinspired research purposes.

First, Arias-Méndez and colleagues [1] present bioinspired algorithms using graph data structures to treat the problem of metabolic pathway pairwise comparison, a computationally complex task. Their promising results provide an innovative perspective towards the study of metabolic pathways by computational means, useful for future research on the topic.

Next, the group led by Olaizola [2] provide a model of business leadership through a biomimetic approach that considers nature as a model, measure, and mentor. Their proposal defines a biomimetic leader from the point of view of the characteristics of biomimetic organizations. They reviewed the main leadership styles analyzed in the recent literature about management and extracted characteristics that will adapt to the biomimetic leadership model, thus obtaining the traits of a biomimetic leader validated by an expert panel. Their work brings a refreshing perspective to the field of business management, particularly to the issue of leadership.

Zumbado-Corrales and Esquivel-Rodríguez [3] present a bio-inspired computation optimization method, an Evolutionary-Optimized Segmentation algorithm, that iteratively improves baseline segments obtained from a classical approach called watershed segmentation. Moreover, they included a cost function based on an ideal segmentation classifier trained as part of their development, which uses basic structural information available to scientists, such as the number of expected units, volume, and topology. As a result, it is proven that a basic initial segmentation with the additional information allowed the evolutionary method to find better segmentation results compared to the baseline generated by the watershed. Their contribution has implications for research on optimization and allows future work to consider their bioinspired algorithm as part of new developments.

Furthermore, Solís and Rojas-Herrera [4] contribute to disease prevention in coffee, forests, and crop fields. They review possible bioinspired machine learning and meteorological and temporal models to predict leaf wetness duration (LWD), an indicator of risk of disease within a plantation. As a result, and based on data collected during their own field work, it is demonstrated that for LWD modeling, it is not convenient to aggregate records at a daily level; performance improves when the records are collected at intervals of 15 min.

Then, Scerrato’s group [5] considers, from an innovative bioinspired perspective, an artificial graft typical in the bone reconstruction surgery with the same microstructure of the bone living tissue and examines the interaction between these two phases, namely bone and the graft material. With the help of a simple example, it is shown that the proposed model can be used as a graft design tool. In fact, an optimization of the characteristics of the implant can be carried out by numerical investigations. As a result, they conclude that the size of the graft considerably influences the interaction between bone tissue and artificial bio-resorbable material and the possibility that the bone tissue might partially substitute the foreign graft for better bone healing.

Finally, Coto-Jiménez [6] presents a new bioinspired approach to postfiltering synthesized voices with the application of discriminative postfilters, with several long short-term memory (LSTM) deep neural networks. His work analyses the discriminative postfilters obtained using five voices, evaluated using three objective measures, Mel cepstral distance, and subjective tests. The results indicate the advantages of the discriminative postfilters in comparison with current postfiltering approaches.

It is our hope that the reader finds the contents of this Special Issue insightful and relevant for their own research purposes. As editors, we have enjoyed the variety and originality of the contributions. Furthermore, we had the privilege of interacting with a myriad of researchers from areas around the world and learned a lot about the possibilities and future perspectives of bioinspired approaches towards the resolution of contemporary complex issues.
